# Vicarious efficacy of tirzepatide in a cohabiting couple: An observational case report

**DOI:** 10.1016/j.obpill.2025.100219

**Published:** 2025-10-21

**Authors:** Kieran Eason, Claire Feeney, Tim Killeen

**Affiliations:** a Easons Pharmacy, Wilnecote, Tamworth, UK; b Imperial Healthcare NHS Trust, London, UK; c Specialist in Pharmaceutical Medicine, Niederweningen, Zurich, Switzerland

**Keywords:** Tirzepatide, Obesity pharmacotherapy, Case report, Vicarious efficacy, Type 2 diabetes, Household dynamics

## Abstract

**Introduction:**

Tirzepatide, a dual glucose-dependent insulinotropic polypeptide (GIP) and glucagon-like peptide-1 (GLP-1) receptor agonist, produces substantial weight loss and glycaemic improvement in people with and without type 2 diabetes mellitus (T2DM). Obesity pharmacotherapy may influence household behaviours and indirectly affect untreated cohabitants, but no prior report has described such an effect.

**Patient main concerns and findings:**

A cohabiting couple presented with obesity. The female partner (Patient A; no diabetes) sought weight loss therapy; the male partner (Patient B) had long-standing T2DM managed with insulin. Both partners were motivated to lose weight but had previously been unsuccessful through diet alone.

**Primary diagnoses interventions and outcomes:**

Patient A began tirzepatide through a pharmacy-supervised weight management programme (escalated to 5 mg weekly). Patient B did not receive any pharmacotherapy but lived in the same household. Over 32 weeks, Patient A lost >30 % of baseline weight. Patient B lost 13 % of baseline weight, HbA1c fell from 9.5 % to 6.1 %, and insulin requirements declined by approximately 70 %. These outcomes paralleled those reported in tirzepatide clinical trials despite absence of medication in Patient B.

**Conclusion:**

This case illustrates potential household-level or “vicarious” efficacy of anti-obesity pharmacotherapy. Environmental and behavioural changes in one partner may yield indirect metabolic benefits for untreated cohabitants. Recognition of such effects could inform patient counselling, cost-effectiveness assessments, and future research into family-based obesity care.

## Graphical abstract

**Figure:**
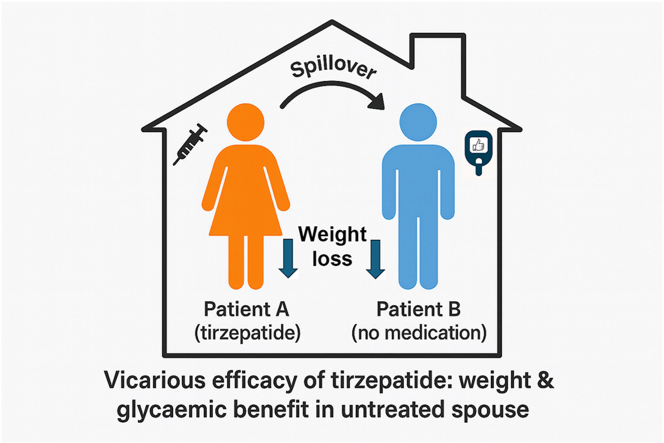

## 1 Introduction

Tirzepatide, a dual glucose-dependent insulinotropic polypeptide (GIP) and glucagon-like peptide-1 (GLP-1) receptor agonist, produces substantial weight loss and glycaemic improvement in people with and without type 2 diabetes mellitus (T2DM) [[1], [2], [3]].

Evidence from lifestyle and bariatric studies shows that weight loss in one partner can influence untreated spouses, sometimes beneficially and sometimes adversely [[4], [5], [6], [7]]. These observations align with evidence that weight-related behaviours cluster within households and social networks, reflecting both shared environments and interpersonal influences [8]. However, no case report has yet described such vicarious efficacy with pharmacotherapy. We report a cohabiting couple in which tirzepatide treatment in one partner coincided with significant weight loss and glycaemic improvement in the untreated spouse.

In the United States (Zepbound®) and in Europe/Switzerland (Mounjaro®), tirzepatide is initiated at 2.5 mg once weekly for 4 weeks and escalated in 2.5 mg steps at ≥4-week intervals, with recommended maintenance doses of 5, 10, or 15 mg once weekly [1,9].

## 2 Case presentation

### 2.1 Patient information

Patient A (index case) was a woman in her late 60s with lifelong obesity but no history of metabolic disease. She was retired from a healthcare career and primarily responsible for household food purchasing and preparation. Previous attempts at weight loss included calorie-restricted diets, commercial programmes, and intermittent joint dieting with her spouse, none of which produced durable reductions below a BMI of 30 kg/m^2^.

Patient B (cohabiting spouse) was a man in his early 70s with obesity, type 2 diabetes mellitus (T2DM) of more than 20 years’ duration, hypertension, and hypercholesterolaemia. His medical history also included statin-associated pancreatitis and autoimmune uveitis. He had made repeated unsuccessful dieting attempts and described difficulty adhering to lifestyle advice. At baseline, he was on basal insulin, metformin, antihypertensive therapy, and ezetimibe.

Both patients reported long-term alcohol abstinence. Patient A described consuming >5 cans of diet cola daily and episodes of compulsive snack-food purchasing. Physical activity was limited for both, comprising mainly dog walking.

### 2.2 Clinical findings

• Patient A: weight 91.0 kg, BMI 34.4 kg/m^2^, waist circumference elevated and hypertension (controlled with amlodipine). No history of diabetes, or major comorbidities.
• Patient B: weight 98.8 kg, BMI 31.3 kg/m^2^, poorly controlled T2DM (HbA1c 9.5 %), mean blood pressure 147/75 mmHg, on insulin and oral therapy.

### 2.3 Timeline

Key events for both patients are summarised in Table 1.

**Table 1:** Timeline of events in Patients A and B.

Timepoint	Patient A (tirzepatide, no T2DM)	Patient B (no tirzepatide, T2DM)
Baseline	Weight 91.0 kg (BMI 34.4); longstanding diet cola habit; limited physical activity. Controlled hypertension.	Weight 98.8 kg (BMI 31.3); HbA1c 9.5 %; BP 147/75; on basal insulin (52 U/day), metformin, antihypertensives, ezetimibe.
Week 0	Enrolled in pharmacy-led programme; started tirzepatide 2.5 mg weekly.	Continued standard diabetes care; no new interventions due to history of pancreatitis (contraindication for GLP-1 agonists).
Week 12	Dose titrated to 5 mg; complete cessation of diet cola; reduced snack purchases.	Portion sizes reduced and healthier foods introduced at home.
Week 24	Weight 72.5 kg (BMI 27.4, −20.3 %); no adverse events.	Weight 88.0 kg (BMI 27.9, −10.9 %); HbA1c 6.1 %; basal insulin ≈20 U/day; BP 105/60.
Week 32	Weight 64.4 kg (BMI 24.4, −29.2 %); entered normal BMI range.	Weight 85.6 kg (BMI 27.1, −13.4 %); HbA1c stable at 6.1 %; basal insulin 16 U/day; metformin dose halved.

### 2.4 Diagnostic assessment

Patient A's baseline assessment was conducted as part of a structured pharmacy-led weight management programme, including measurement of body weight, BMI, and waist circumference. No additional laboratory investigations were indicated.

Patient B's assessments were derived from routine diabetes care. Baseline HbA1c was 9.5 %. Blood pressure, lipid profile, and renal/hepatic function were within the ranges expected for his comorbid conditions (Table 1).

### 2.5 Therapeutic intervention

Patient A enrolled in a community pharmacy-led weight management service. Tirzepatide was initiated at 2.5 mg once weekly and escalated stepwise in line with UK prescribing information [1]. The lowest effective dose was maintained (5 mg in this case). Counselling focused on adherence to injection technique, safe storage and disposal, and combining pharmacotherapy with structured lifestyle advice. Lifestyle recommendations included portion control, avoidance of calorie-dense foods, meal planning, and continued alcohol abstinence. From the outset of treatment, Patient A reported immediate, complete cessation of a lifelong diet cola habit and reduced purchasing of snack foods.

Patient B did not receive tirzepatide or any other GLP-1 receptor agonist, as his prior history of pancreatitis was considered a contraindication by the diabetes nurse service. His pharmacological management continued unchanged with basal insulin, metformin, antihypertensive therapy, and ezetimibe. No new lifestyle programme was prescribed. However, his dietary intake was indirectly modified through household changes introduced by Patient A: reduced caloric availability, healthier food purchases, and smaller portion sizes. No new medications or acute psychosocial stressors were reported during the observation period.

### 2.6 Follow-up and outcomes

**Fig. 1:**
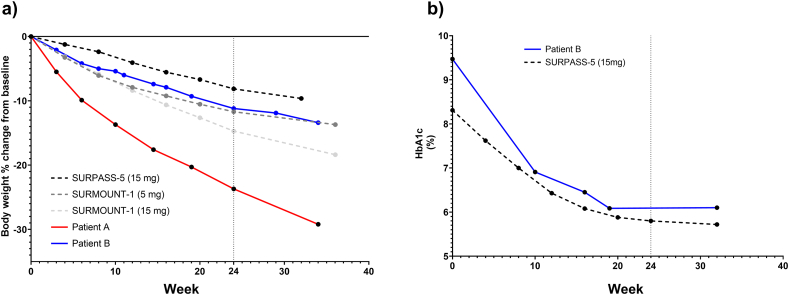
Body weight and HbA1c trajectories for Patients A and B compared with pivotal tirzepatide trials. The vertical dotted line marks week 24, corresponding to the comparator period from SURMOUNT-1 and SURPASS-5 and the end of the initial 24-week pharmacy programme. Trial datapoints were digitised from the EMA European Public Assessment Report (SURMOUNT-1) and Dahl et al. (SURPASS-5) using WebPlotDigitizer (https://automeris.io/wpd/). ***Left panel (a):*** Percentage change in body weight from baseline. Patient A (solid red line; tirzepatide 5 mg via pharmacy programme, no diabetes) and Patient B (solid blue line; untreated spouse with T2DM on insulin) are shown alongside mean trajectories from SURMOUNT-1 (dashed grey lines; tirzepatide 5 mg in medium grey, 15 mg in light grey) and SURPASS-5 (dashed black line; tirzepatide 15 mg, trial mean baseline 95.3 kg). Patient A achieved >20 % loss by 24 weeks and >30 % by 32 weeks. Patient B lost ≈11 % by 24 weeks and ≈13 % by 32 weeks, broadly aligning with trial outcomes despite absence of pharmacotherapy. ***Right panel (b):*** HbA1c trajectories. Patient B (solid blue) is compared with the SURPASS-5 15 mg arm (dashed black; trial mean baseline HbA1c 8.31 %). Patient B's HbA1c fell from 9.5 % at baseline to 6.1 % at 24 weeks and remained stable at 32 weeks, despite a ≈70 % reduction in insulin dose and halving of metformin. (For interpretation of the references to colour in this figure legend, the reader is referred to the Web version of this article.)

By week 24, Patient A's weight had decreased to 72.5 kg (BMI 27.4 kg/m^2^), a reduction of 20.3 %. At week 32 she had lost a further 8.1 kg, reaching 64.4 kg (BMI 24.4 kg/m^2^), thereby entering the normal BMI range. She reported no adverse events and continued to adhere to smaller, healthier meals. Her trajectory exceeded mean weight-loss outcomes reported at similar timepoints in SURMOUNT-1, where adults without T2DM achieved approximately 15 % loss at 26 weeks (Fig. 1) [2].

Patient B, who did not receive pharmacotherapy, weighed 98.8 kg at baseline (BMI 31.3 kg/m^2^). By week 24, his weight had fallen to 88.0 kg (BMI 27.9 kg/m^2^), a reduction of 10.9 %. HbA1c improved markedly from 9.5 % at baseline to 6.1 %, with basal insulin reduced from 52 to approximately 20 units/day and blood pressure settling into the normotensive range (105/60 mmHg). By week 32 his weight had further declined to 85.6 kg (−13.4 %), his HbA1c remained stable at 6.1 %, and insulin requirements decreased to 16 units/day (≈70 % reduction overall), despite halving his metformin dose. His outcomes paralleled trajectories seen in the active treatment arms of SURPASS-5, in which patients with T2DM on insulin achieved mean weight reductions of 5–9 % and HbA1c reductions of 1.2–2.3 points by 40 weeks (Fig. 1) [3].

Both patients attributed their changes primarily to modified food purchasing, smaller portions, and reduced caloric availability within the household. Physical activity levels increased only modestly.

## 3 Discussion

This case demonstrates parallel, clinically meaningful weight loss and metabolic improvement in a cohabiting couple, despite only one partner receiving tirzepatide. Patient A, treated within a structured pharmacy-led programme, achieved >20 % weight loss at 24 weeks and nearly 30 % at 32 weeks, exceeding typical outcomes in SURMOUNT-1, where adults without T2DM lost a mean of ≈15 % at 26 weeks [2]. Patient B, with long-standing T2DM, lost ≈11 % of body weight, normalised HbA1c from 9.5 % to 6.1 %, and reduced basal insulin by ≈70 %, aligning with outcomes observed in tirzepatide-treated patients in SURPASS-5, where mean HbA1c reductions ranged from 1.2 to 2.3 % and mean weight loss 5–9 % at 40 weeks [3]. Remarkably, he achieved these results without pharmacotherapy, underscoring the clinical importance of this household-level effect.

To our knowledge, no prior case report has documented vicarious efficacy of an obesity pharmacotherapy. Evidence from lifestyle and bariatric studies demonstrates that untreated spouses often experience secondary effects of their partner's intervention. In the DIRECT-Spouse trial, untreated partners experienced modest weight loss over two years [4,7]. Following bariatric surgery, spouses may also benefit: Fawcett et al. reported weight loss and healthier eating in cohabitants one year post-Roux-en-Y gastric bypass [6]. Conversely, Madan et al. found some spouses gained weight by consuming patients' uneaten portions [7]. Together, these reports highlight that shared environments can amplify or undermine weight-loss interventions, but pharmacotherapy has not previously been shown to generate such effects.

Several mechanisms may explain Patient B's outcomes. Patient A, as the household food gatekeeper, reduced caloric availability, eliminated snack foods, and downsized portions, directly altering dietary intake. Both partners reported modest increases in activity and continued their long-standing alcohol abstinence, suggesting dietary modification was the dominant driver. Psychological mechanisms may also have contributed: Patient B was unblinded to his spouse's treatment, and her rapid progress may have provided motivation. The patient himself believes his success was primarily driven by passive adherence to a changed household food environment and, to a lesser extent, psychological motivation arising from his spouse's success.

These findings have implications for clinical care and policy. Clinicians should consider household context when counselling patients on pharmacotherapy, as family members may derive secondary benefits. From an ethical perspective, such spill-over effects highlight the need to respect autonomy and consent while recognising potential indirect benefits. From a health-economic perspective, indirect improvements in untreated cohabitants could enhance the value proposition of pharmacotherapy. Current cost-effectiveness models typically focus on the treated individual and do not account for wider household effects, potentially underestimating true benefit. Future modelling could incorporate these ripple effects, especially in conditions like T2DM where modest weight loss yields major cost savings [10].

Beyond adult households, these findings may also have implications for the distinct challenge posed by paediatric obesity, which remains a major global public health burden associated with early-onset metabolic disease and long-term cardiometabolic risk. Parents are powerful determinants of the home food environment, acting as dietary gatekeepers whose own weight change and modelling behaviours strongly influence their children's eating patterns and weight trajectories [[11], [12], [13]]. If pharmacotherapy in one or both parents induces sustained improvements in household diet quality, similar vicarious benefits could potentially extend to children and adolescents. This may merit prospective study, particularly as anti-obesity medications become increasingly prescribed to adults of child-rearing age.

This case highlights the need for future research on how pharmacotherapy programmes might harness household spill-over effects, whether couples-based or family education could enhance outcomes, and which household or interpersonal factors moderate these vicarious responses. Such work would extend the emerging literature on family-based obesity interventions into the pharmacotherapy era.

## 4 Conclusion

This case illustrates a novel observation of vicarious efficacy in obesity pharmacotherapy. Initiation of tirzepatide in one partner was associated with clinically significant weight loss and metabolic improvement in the untreated spouse with T2DM. The parallel trajectories compared with pivotal trial outcomes highlight the potential for household-level effects of obesity treatment. While causality cannot be inferred from a single case, the finding suggests that clinicians and researchers should consider the shared environment when evaluating pharmacotherapy outcomes.

### 4.1 Key clinical messages

• Obesity pharmacotherapy may indirectly benefit untreated cohabitants through environmental and behavioural changes.
• Clinicians should consider household context when counselling patients starting treatment.
• Indirect benefits may enhance cost-effectiveness and justify further household-level research.

## 5 Patient perspectives

### 5.1 Patient A

“I am grateful this treatment became available in my lifetime and that it is helping me to become fitter as I get older. After a lifetime of failed attempts at weight loss through slimming clubs, I feel that with this treatment I can finally succeed. The one-to-one support from the pharmacist has been very helpful and reassuring. Sometimes I feel I have taken an easy option, and I am still surprised when people comment on how much weight I have lost and how different I look.”

### 5.2 Patient B

“I have been overweight all my life and tried dieting hundreds of times. Because I had pancreatitis in the past, I was not able to take weight loss drugs. I am now lighter than I have been for 50 years. Since my partner started injections, dieting has been much easier. She does most of the shopping and has stopped buying treats, so there are healthier foods in the house and no takeaways. That has made it much easier for me to succeed. I do worry a little about what will happen if she stops the treatment.”

## Ethical declarations

Written informed consent was obtained from both patients for publication of this case report and accompanying figure. Ethical approval was not required. All data underlying the results are included in this article.

## CRediT author contributions

Conceptualisation: TK, KE, Investigation: KE, Writing – original draft: TK, Writing – review and editing: KE, CF, TK, Visualisation: TK. KE, CF and TK approved the final submission and publication.

## Declaration of generative AI in scientific writing

During the preparation of this work, the authors used ChatGPT (OpenAI, San Francisco, CA, USA) to assist with restructuring to match journal formatting requirements and graphical abstract design. After using this tool, the authors reviewed and edited the content as needed and take full responsibility for the content of the publication.

## Funding

There was no specific funding for the preparation of this manuscript.

## Declaration of competing interests

KE is superintendent pharmacist and owner of Easons Pharmacy. Patient A's treatment was carried out as part of the private pharmacological weight-loss program offered by the pharmacy. The clinical management of this patient was remunerated as part of the pharmacy business.

CF has no disclosures relating to the contents of this manuscript.

TK is an employee of Pfizer AG., the Swiss affiliate of Pfizer Inc., a global biopharmaceutical company. He is also a shareholder of Pfizer Inc. Pfizer was not involved in any aspect of the conception, drafting or decision to submit this manuscript for publication.

The authors declare that they have no other competing interests relevant to this case report.
